# The influence of nativity/birthplace, neighborhood cohesion, and duration lived in the neighborhood on psychological distress

**DOI:** 10.1016/j.jadr.2024.100798

**Published:** 2024-05-03

**Authors:** Lohuwa Mamudu, Jolyna Chiangong, Michael Curry, Archana J. McEligot, Hadii M. Mamudu, Faustine Williams

**Affiliations:** a Department of Public Health, College of Health and Human Sciences, California State University, Fullerton, CA, USA; b Division of Intramural Research, National Institute on Minority Health and Health Disparities, National Institutes of Health, Bethesda, MD, USA; c Information Management Services Inc., Calverton, MD, USA; d Center for Cardiovascular Risk Research, East Tennessee State University, Johnson City, TN, USA; e College of Public Health, East Tennessee State University, Johnson City, TN, USA

**Keywords:** Psychological distress, Nativity/birthplace, Neighborhood cohesion, Length of residence in neighborhood, Years lived in neighborhood

## Abstract

**Introduction:**

Nativity/birthplace and neighborhood cohesion are potential contributing factors to psychological distress. This study explores the impact of nativity/birthplace and neighborhood cohesion on moderate-severe psychological distress among United States (US) adults, considering the duration lived in a neighborhood.

**Methods:**

Using the 2013–2018 National Health Interview Survey data, we conducted a stratified analysis based on years lived in the neighborhood (≤10 years [*n* = 96,175] and >10 years [*n* = 68,187]). Bivariate chi-square tests and multivariable logistic regression models were used to assess the statistical differences and associations between moderate-severe psychological distress and nativity/birthplace, and neighborhood cohesion, while adjusting for other covariates.

**Results:**

Individuals with 10 years or less of residence reported higher levels of moderate-severe psychological distress than those with more than 10 years (22.3 % vs. 18.1 %). Low or medium neighborhood cohesion, regardless of duration of residence, was associated with significantly higher odds of moderate-severe psychological distress compared to high cohesion. Foreign-born individuals had higher odds of psychological distress after more than 10 years in a neighborhood, although this difference was not statistically significant. However, they had lower odds of psychological distress after 10 years or less in the neighborhood compared to US-born individuals. Similarly, the interaction of foreign-born status and 10 years or less of residence in a neighborhood showed decreased odds of psychological distress.

**Conclusions:**

These findings underscore the importance of strong social cohesion in neighborhoods for positive mental well-being. Establishing community initiatives to enhance neighborhood social cohesion is crucial.

## 1. Introduction

Psychological distress is a common mental health problem with increasing prevalence in the United States (US) (Gullett et al., 2022; Weissman et al., 2015). Among US adults aged 18 years and older, 3.4 % experienced serious/severe psychological distress, including 3.9 % of women and 2.8 % of men (Weissman et al., 2015). Psychological distress is characterized by emotional and mood disorders (i.e., depression, anxiety, stress, nervousness) which can impact an individual’s ability to effectively respond to everyday demands of life (Arvidsdotter et al., 2016; Gullett et al., 2022; Ismail et al., 2020). Psychological distress has been associated with a lack of harmony or congruence between one’s sense of self and their ideal self. (Arvidsdotter et al., 2016). This discrepancy may also contribute to a gradual reduction in existential capacities or the ability to meaningfully engage with life’s challenges and uncertainties. Several factors have been reported to contribute to the development of psychological distress, including neighborhood cohesion (Gullett et al., 2022; Rios et al., 2012), nativity/birthplace (Dallo et al., 2013), sociodemographic (Agrawal et al., 2015) and socioeconomic (Erdem et al., 2016) characteristics.

Neighborhood cohesion represents the psychological sense of community, attraction to neighborhood, and social interaction within a neighborhood (Buckner, 1988). The extent of cohesiveness in a neighborhood may impact individuals’ physical and mental health. For instance, high perceived neighborhood cohesion was reported to directly impact better mental health and shield against the effect of deprivation on health (Fone et al., 2007). Similarly, Erdem et al. (2016) noted that high social neighborhood cohesion was associated with lower psychological distress (Erdem et al., 2016), as individuals in a socially cohesive neighborhood have a strong willingness to help each other (Kawachi and Berkman, 2000). Neighborhood cohesion may also serve as a protective factor for mental health and a buffer against the effects of daily stressors such as low socioeconomic status (Rios et al., 2012; Robinette et al., 2013). In contrast, a study by Ruiz et al. (2018) found that adults with low perceived social cohesion in their neighborhood had less depressive symptoms than their counterparts with high perceived social cohesion as they aged (Ruiz et al., 2018).

Additionally, the place or region of birth, also known as nativity, may also have an impact on mental health. Dallo et al. (2013) found that foreign-born non-Hispanic Whites from the Middle East were more likely to report psychological distress than US-born non-Hispanic Whites (Dallo et al., 2013). Nativity status can also exacerbate the negative effects of discrimination on mental health, as reported among US-born individuals (Yip et al., 2008). Evidence from North America has revealed higher odds of psychological distress and other mental health disorders among migrants (Breslau et al., 2009; Islam et al., 2014). The experience of migration and resettling in a new place presents potential acculturation challenges, such as culture shock, unemployment, financial and status changes, disruption of household and family dynamics, language difficulties, attitudes of the receiving community, racism, and stereotyping (Dow, 2011). Dow (2011) reported that immigrants face mental difficulties due to the myriad of stressors, obstacles, and challenges upon arrival in a new environment within the US (Dow, 2011). The influence of nativity (foreign-born vs. US-born) on the prevalence of mental health disorders has shown varying results across different racial groups (Alegría et al., 2008; Takeuchi et al., 2007). For instance, Hispanic/Latino subjects were reported to be at lower risk of most mental health disorders than non-Hispanic/Latino Whites, whereas US-born Hispanics/Latinos were at higher risk than Hispanic/Latino immigrants (Alegría et al., 2008). A study on mental health of Asian Americans showed that they have lower rates of mental health issues compared to the general population, and nativity influenced the pattern of this outcome (Takeuchi et al., 2007). However, in another study, nativity-related immigration stress was found to be significantly positively associated with psychological distress, as reported by Torres et al. (2013). The authors reported that migrating to the US was associated with increased psychological distress among Puerto Rican and Cuban women (Torres and Wallace, 2013).

Several factors, including sociodemographic characteristics and socioeconomic disparities, have been shown to impact levels of psychological distress (Erdem et al., 2016). Individuals with a low income (Lorant et al., 2003), low level of education (Peyrot et al., 2013) or unemployment (Fryers et al., 2005) are at high risk of mental health problems. Studies have found that the prevalence of psychological distress is higher just after migration, influenced by sociodemographic characteristics and socioeconomic factors (Agrawal et al., 2015; Cleary and Mechanic, 1983). For example, women consistently face a higher risk of psychological distress compared to men (Cleary and Mechanic, 1983). Recent literature has also revealed differences in the relationship between neighborhood cohesion and psychological distress when considering different racial/ethnic backgrounds (Gullett et al., 2022). Another study also reported that ethnic identity has a significant effect on migrant distress (Nesdale et al., 1997).

The extent of neighborhood cohesion can be influenced by how long an individual has lived in a neighborhood, which can impact their psychological distress. For instance, Quillian (2003), examining the long-term dynamics of entry and exit from poor neighborhoods reported that Black African Americans were more likely to live in a poor neighborhood for more than 10 years than Whites, which was often influenced by low-income (Quillian, 2003), which is a determinant of psychological distress. Further, a study conducted on Canadians also found that high psychological distress was more associated with low-income populations (Caron and Liu, 2011). Additionally, Jurado et al. (2017), in an international review on migrants’ psychological distress, reported that the duration of residence in the receiving country was associated with distress (Jurado et al., 2017). Although some studies have examined the association between nativity and neighborhood cohesion with psychological distress, few studies have investigated this relationship in a large nationally representative population-based sample, and no studies have investigated this relationship stratified by length of residence or years in their respective neighborhood. Therefore, this study aims to examine nativity/birthplace and neighborhood cohesion association with moderate-to-severe (moderate-severe) psychological distress stratified by duration of residence or years lived in the present neighborhood, while adjusting for sociodemographic, socioeconomic status, and health-related behavioral risk factors. We hypothesize that nativity/birthplace and neighborhood cohesion would influence individuals’ likelihood of experiencing moderate-severe psychological distress and that the duration of residence may modify this association.

## 2. Materials and methods

### 2.1. Data and study design

#### 2.1.1. Data source

The present study utilized population-based cross-sectional household interview survey data of US adults obtained from the National Health Interview Survey (NHIS), spanning from 2013 to 2018 (IPUMS Health Surveys: National Health Interview Survey). NHIS is a primary data collection program of the National Center for Health Statistics (NCHS), which is part of the Centers for Disease Control and Prevention (CDC) and is considered the principal source of data on the health of the civilian noninstitutionalized population in the US. Institutional Review Board (IRB) approval was not required as NHIS data used in this study is publicly available and the de-identified data are accessible via the NCHS CDC website (IPUMS Health Surveys: National Health Interview Survey).

#### 2.1.2. Sample selection

The initial sample consisted of 190,113 individuals. After excluding individuals with missing responses and nonresponse, 25,751 individuals were listwise deleted from the analysis. The final analysis included an unweighted sample of 164,362 individuals with complete information on psychological distress, nativity/birthplace, and neighborhood cohesion, stratified by duration of residence or years lived in the current neighborhood (i.e., ≤10 years; *n =* 96,175; >10 years; *n =* 68,187). This stratification is consistent with previous studies investigating the duration lived in the US, particularly those focusing on immigrants or foreign-born individuals (Breslau et al., 2007; Cook et al., 2009). A study on immigration and mental health reported that years in the US moderate discrimination, stress, and mental health (Alegría et al., 2017). Additionally, Uretsky and Mathiesen (2007), reported that the health advantage foreign-born individuals enjoy upon arrival in the US slowly decreases and becomes uniform with US-born persons as the number of years lived in the US increases, especially beyond 10 years.

### 2.2. Measures

#### 2.2.1. Outcome: psychological distress

The outcome variable of this study is psychological distress based on the Kessler scale (K-6). Individuals were asked the following questions: “During the past 30 days, how often did you feel: sad, nervous, restless, hopeless, that everything was an effort, or worthless?” Response choices are based on 5-point Likert-scale ranging from “none of the time” (0) to “all of time” (4). The scores of the six questions are then summed, yielding a minimum score of 0 and a maximum score of 24. Low scores indicate low levels of psychological distress and high scores indicate high levels of psychological distress [i.e., 0–4= none/low psychological distress; 5–12= moderate psychological distress; & ≥13= serious/severe psychological distress] (Fushimi et al., 2012; Kessler et al., 2002, 2003). For the purpose of this study, we dichotomized the K-6 classification as none/low for a K6 score of <5 as having no/low psychological distress, and moderate-severe for a K-6 score of ≥5 as having psychological distress.

#### 2.2.2. Exposures: neighborhood cohesion and nativity/birthplace

The major exposures under consideration included neighborhood cohesion and nativity/birthplace. For neighborhood cohesion, NHIS asked individuals to agree to the following four statements using a Likert response scale of “definitely agree = 1″, “somewhat agree = 2″, “somewhat disagree = 3″, “definitely disagree = 4″: “(1) this is a close-knit neighborhood, (2) there are people you can count on in this neighborhood, (3) people in this neighborhood can be trusted, and (4) people in this neighborhood help each other out.” Each Likert scale response was reverse coded and calculated as the sum of the four response items ranging from 4 to 16. Tertile cut-points were used to classify neighborhood cohesion scores as follows: 4–11 for low cohesion, 12–14 for medium cohesion, and 15–16 for high cohesion (Alhasan et al., 2020; Quinn et al., 2019; Young et al., 2018). The cut-points were based on the weighted distribution of neighborhood cohesion among all participants. For nativity/birthplace, individuals were classified as either US-born or foreign-born/immigrants. Individuals born in one of the 50 states, or the District of Columbia (D.C.), were considered US-born. Persons born outside the US or its territory (including Puerto Rico, Northern Mariana Islands, Guam, US Virgin Islands, and American Samoa) were grouped as foreign-born/immigrants (Clarke et al., 2019; Krieger et al., 2011).

#### 2.2.3. Covariates

Other potential exposures or covariates included sociodemographic variables are: sex (i.e., male or female), race/ethnicity (non-Hispanic Asian, non-Hispanic Black, Hispanic/Latino, non-Hispanic White, or Other/multi-racial), marital status (single/never married, married/living with partner, divorced/separated, or widow/widower). Socioeconomic variables included education attainment (less than high school, high school, some college, or college/graduate), employment status (employed, or unemployed), and family income (i.e., <$25,000, $25,000-$49,000, $50,000-$74,000, or ≥$75,000). Health and behavioral/lifestyle risk variables were as follows: alcohol drinking status (i.e., lifetime abstainer (<12 drinks in life), former (no drinks past year), or current (1+ drinks past year), smoking status (never, former, or current), and body mass index (BMI) <18.5, underweight, 18.5–24.99, healthy weight, 25–29.9, overweight or ≥30, obesity.

### 2.3. Statistical analysis

All analysis samples were weighted to incorporate the NHIS complex survey design, accounting for the strata, primary sampling units, and survey weights to ensure estimates were nationally representative (National Center for Health Statistics. Centers for Disease Control and Prevent). Stratified by the duration lived in the neighborhood, we estimated the weighted frequencies of the sample characteristics by psychological distress, nativity/birthplace, neighborhood cohesion and other sociodemographic, socioeconomic, and health behavior factors (see Table 1). We used Rao-Scott chi-square tests for bivariate statistics to assess differences in moderate-severe psychological distress among the levels of the exposures (i.e., nativity/birthplace and neighborhood cohesion) and covariates (see Table 1). Next, we conducted two multivariable logistic regression analyses (Model I and Model II, see Table 2), to examine the association between the exposures and moderate-severe psychological distress, adjusting for covariates. In Model I, we examined the odds of moderate-severe psychological distress among individuals who lived in the present neighborhood for <10 years, while Model II examined the odds of moderate-severe psychological distress among those who lived in the present neighborhood for >10 years. Further we conducted an interaction model to examine the length of residence in a neighborhood as an effect modifier of nativity/birthplace and neighborhood cohesion influence on psychological distress (see Table 3). We summarized and assessed the statistical significance of the parameter estimates based on adjusted odd ratios (AOR), 95 % confidence intervals (95 % CI) of AOR, and p-values of <0.05 significance levels. All statistical analyses were conducted using the SUDAAN version 11.0.3 statistical software.

## 3. Results

### 3.1. Sample characteristics and bivariate differences in moderate-severe psychological distress by duration in neighborhood

The largest subsample consisted of individuals who had lived in the neighborhood for at most 10 years, comprising 58.5 % of the sample, while the remaining 41.5 % had lived in the neighborhood for more than 10 years. Among the total participants, approximately 80 % reported none/low psychological distress, while 20.5 % reported moderate-severe psychological distress. The subpopulation analysis showed that 22.3 % of individuals who had lived in the neighborhood for 10 years or less reported moderate-severe psychological distress, compared to 18.1 % of those who had lived for more than 10 years and reported moderate-severe psychological distress. Most individuals who had lived in the neighborhood for ≤10 years reported low neighborhood cohesion (36.4 %), while those who had lived for >10 years reported high neighborhood cohesion (40.8 %). Additionally, the majority of the sample were US-born natives (≤10 years= 79.1 % vs. >10 years= 86.6 %), married/living with a partner (≤10 years= 60.5 % vs. >10 years= 62.5 %), non-Hispanic White (≤10 years= 60.9 % vs. >10 years= 72.3 %), had completed college/graduate education (≤10 years= 33.5 % vs. >10 years= 30.3 %), employed (≤10 years= 68.3 % vs. >10 years= 53.6 %), had a family income of ≥$75,000 (≤10 years= 38.3 % vs. >10 years= 45.0 %), were current drinkers (≤10 years= 69.6 % vs. >10 years= 62.8 %), and never smokers (≤10 years= 63.6 % vs. >10 years= 60.8 %). Also, most individuals who lived in the neighborhood for ≤10 years had normal BMI/healthy weight (35.3 %), while most of those who lived for >10 years were overweight (35.7 %). There was a statistically significant (*p* < 0.0001) difference in the levels of psychological distress, neighborhood cohesion, and nativity/birthplace among individuals living in a neighborhood for ≤10 years and >10 years. This was similar for all the covariates, except sex. See Table 1 for details.

### 3.2. Multivariable logistic regression of moderate-severe psychological distress for 10 years or less and more than 10 years residence in current neighborhood

Table 2 shows results on the relationship between neighborhood cohesion, as well as nativity/birthplace with moderate-severe psychological distress stratified by duration of residence (≤10 years and >10 years in a neighborhood). For those residing in a neighborhood for 10 years or less (Model I), participants who reported low and medium cohesion were significantly more likely to experience moderate-severe psychological distress (AOR= 2.01, 95 % CI: 1.88–2.15 and AOR= 1.44, 95 % CI: 1.35–1.54, respectively), compared to those with high neighborhood cohesion. In terms of nativity/birthplace, foreign-born individuals had lower odds of experiencing moderate-severe psychological distress (AOR= 0.63; 95 % CI: 0.45–0.82) relative to US-born individuals. Males (AOR= 0.73; 95 % CI: 0.69–0.76) were less likely than females to report moderate-severe psychological distress. Similarly, being married/living with a partner was associated with lower odds of psychological distress (AOR= 0.82; 95 % CI: 0.77–0.87), as well as being widowed (AOR= 0.72; 95 % CI: 0.65–0.80) compared to being single/never married among those who had lived in their present neighborhood for no more than 10 years. All socioeconomic factors, such as education, employment status, and family income, were significantly associated with higher odds of moderate-severe psychological distress. For instance, individuals who reported having completed less than high school, high school, or some college had increased odds of experiencing moderate-severe psychological distress (AOR= 1.27; 95 % CI: 1.17–1.39 vs. AOR= 1.11; 95 % CI: 1.04–1.19 vs. AOR= 1.16; 95 % CI: 1.10–1.23, respectively) compared to those with college/graduate degrees. We also found significant associations between health behavioral risk factors and moderate-severe psychological distress. Underweight individuals (AOR= 1.38; 95 % CI: 1.19–1.61) or obese individuals (AOR= 1.35; 95 % CI: 1.27–1.43) had higher odds of moderate-severe psychological distress compared to normal weight individuals. In terms of alcohol drinking status, former drinkers and current drinkers had increased odds of experiencing moderate-severe psychological distress relative to lifetime abstainers (AOR= 1.42; 95 % CI: 1.31–1.54 and AOR=1.38; 95 % CI: 1.29–1.48, respectively). Compared to never smokers, being a former smoker (AOR=1.20; 95 % CI: 1.14–1.27) or current smoker (AOR= 1.84; 95 % CI: 1.74–1.94) was significantly associated with moderate-severe psychological distress.

In Model II (Table 2), individuals who reported low or medium neighborhood cohesion and had lived in their neighborhood for more than 10 years had high likelihood of reporting moderate-severe psychological distress (AOR= 2.06; 95 % CI: 1.90–2.24 and AOR= 1.42; 95 % CI: 1.31–1.55, respectively), compared to those with high neighborhood cohesion. Although not statistically significant, foreign-born individuals had higher odds of experiencing moderate-severe psychological distress (AOR= 1.31; 95 % CI: 0.45–3.79) compared to US-born individuals. Similar to those residing for 10 years or less in a neighborhood, individuals who had lived in a neighborhood for more than 10 years exhibited a consistent pattern of significantly increased or decreased odds of association with moderate-severe psychological distress when considering covariates such as sociodemographic and socioeconomic factors.

### 3.3. Length of residence in current neighborhood as effect modifier of neighborhood cohesion and nativity/birthplace association with moderate-severe psychological distress

The interaction between individuals who experienced low neighborhood cohesion and 10 years or less of residence showed significantly decreased odds of moderate-severe psychological distress compared to those who experienced low cohesion and more than 10 years of residence (AOR= 0.86; 95 % CI: 0.79–0.93). This pattern was similar for individuals who experienced medium neighborhood cohesion and 10 years or less of residence compared to those who experienced medium cohesion and more than 10 years of residence, but it was not statistically significant. Additionally, foreign-born individuals with 10 years or less of residence had significantly decreased odds of moderate-severe psychological distress compared to foreign-born individuals with more than 10 years of residence (AOR= 0.73; 95 % CI: 0.66–0.81). Table 3.

## 4. Discussion

This study is the first to investigate the association of psychological distress with nativity/birthplace and neighborhood cohesion by duration lived in present neighborhood, while controlling for sociodemographic characteristics, socioeconomic status, and health behavior risk factors. The results revealed statistically significant differences in moderate-severe psychological distress based on neighborhood cohesion, nativity/birthplace, and covariates, which align with previous research findings (Agrawal et al., 2015; Dallo et al., 2013; Erdem et al., 2016; Gullett et al., 2022).

We found that, individuals who lived in a neighborhood for 10 years or less, as well as those who lived in a neighborhood for more than 10 years with low or medium neighborhood cohesion, had a higher likelihood of experiencing moderate-severe psychological distress. This finding is consistent with a study by Erdem et al. (2016), which found that high social neighborhood cohesion was associated with low psychological distress among urban adults. Additionally, we found that those who lived in their neighborhood for more than 10 years with low neighborhood cohesion experienced a 5 % higher increased likelihood of moderate-severe psychological distress than those who lived for 10 years or less. Consistently, when interacting neighborhood cohesion with length of residence, we found that experiencing low cohesion for no more than 10 years was associated with lower odds of moderate-severe psychological distress than experiencing low cohesion for more than 10 years . On the other hand, those with medium cohesion and who had lived in their present neighborhood for no more than 10 years experienced higher moderate-severe psychological distress than those who lived for over 10 years. This suggests that the duration of residency in a US neighborhood may have distinct influence on psychological distress, regardless of the level of neighborhood cohesion.

Previous research has also highlighted the role of the duration of residency in a neighborhood and its influence on social bonds and perceptions of neighborhood cohesion. For example, a study on elderly neighbors found that length of residence was a significant positive factor for local friendship (Oh, 2003). Similarly, Pabayo et al. (2020) noted that length of residence in a neighborhood may influence one’s perception of neighborhood social cohesion (Pabayo et al., 2020). Gullett et al. (2022) also considered length of residence as a potential confounder in their study on the associations between neighborhood social cohesion and psychological distress, and found significant results after accounting for this factor (Gullett et al., 2022). These findings highlight how the duration of residency in a neighborhood can interact with neighborhood cohesion to influence psychological distress.

Also, our findings suggest that decreased neighborhood cohesion is associated with increased moderate-severe psychological distress, and the duration of years lived in the neighborhood plays a substantial role in this association. Previous research has shown that neighborhood cohesion can impact physical and mental health outcomes (Mair et al., 2010; Robinette et al., 2018, 2013), with lower neighborhood cohesion being associated with low trust relationships, poorer social support, and less communal connectedness, which can contribute to mental health disorders (Breedvelt et al., 2022; Kim and Kawachi, 2017; Perez et al., 2015; Robinette et al., 2018, 2013). On the other hand, higher neighborhood cohesion is linked to lower daily stressors (Robinette et al., 2013) and increased trust and respect, which are related to better health (Poortinga, 2012). These findings highlight the importance of building strong communities and environments with high neighborhood cohesion as a means to reduce the likelihood of psychological distress.

Furthermore, individuals who have lived in a neighborhood for no more than 10 years and experienced low or medium cohesion may potentially be facing stressors associated with adjusting to a different environment. Studies have shown that migration and pre-migration experiences can have mental health effects, and assimilating into a different culture can have negative effects on mental health and functioning of families (Agrawal et al., 2015). As people experience a short-term stay in a neighborhood, they may encounter issues such as changes in social and personal ties, reconstruction of social networks, movement from one socio-economic system to another, and a shift from one cultural system to another (Kirmayer et al., 2011). These factors may contribute to increased psychological distress.

On the contrary, individuals who have lived in a neighborhood for more than 10 years and experienced low or medium neighborhood cohesion may also serve as a stressor and possibly influence the likelihood of psychological distress. A study on the relationship between chronic and episodic stressors and psychological distress found that exposure to chronic stressors elevates psychological distress levels (Lepore et al., 1997). It is not clear why some individuals may experience low or medium cohesion in their neighborhood despite having lived there for more than 10 years. However, a study on the Swedish population found that psychological distress increases with the duration of residence (Honkaniemi et al., 2020), which aligns with some of our findings. This may help explain why people experience low or medium cohesion in their neighborhood after such a long period of residence. The similarity in the trend of low or medium neighborhood cohesion being associated with an increased likelihood of psychological distress, regardless of differences in duration of stay in a neighborhood, suggests the need for further research to examine the progression of psychological distress as people live in a neighborhood with low or medium cohesion.

Foreign-born individuals were 37 % less likely to report moderate-severe psychological distress having lived in a neighborhood for 10 years or less compared to US-born natives, when considering nativity or birthplace. Similarly, the interaction of foreign-born individuals with at most 10 years of residence was associated with a decreased likelihood of moderate-severe psychological distress compared to that of foreign-born persons with more than 10 years of residence. Although not statistically significant, foreign-born individuals were relatively 31 % more likely than their US-born counterparts to experience moderate-severe psychological distress when they had lived in a neighborhood for more than 10 years. This suggests that as the duration of stay within a neighborhood increases, foreign-born individuals are more likely to experience higher levels of psychological distress. Studies have shown that greater degrees of acculturation are associated with adverse health outcomes including mental health (Alegría et al., 2008; Schwartz et al., 2010). The difference in psychological distress between migrants and natives is greatly influenced by social cohesion, discrimination, and socioeconomic inequalities (Honkaniemi et al., 2020). Additionally, migrants experience worse psychological distress with longer residence, as reported in a study on psychological distress by age at migration and duration of residence in Sweden (Honkaniemi et al., 2020). Further research is warranted to better understand why foreign-born individuals experience an increase in psychological distress as they live longer in a given neighborhood in the US. Further, immigrant support policies on acculturation may help ease the integration of foreign-born individuals into US neighborhoods, improving their psychological health, mental health, and overall well-being.

### 4.1. Limitations

Our study has some limitations. The NHIS program utilizes a cross-sectional survey, making it difficult to establish causal relationships between exposures and psychological distress. Also, different US neighborhoods, including states, cities, counties, etc., may influence individuals’ psychological distress differently, which was not accounted for in this study. Nevertheless, the present study is among the first to investigate a large population-based representative sample, the role of nativity/birthplace and neighborhood cohesion in psychological distress, while accounting for the length of residence or duration lived in the neighborhood. The findings can provide insights and serve as the basis for developing longitudinal and other cohort studies to better understand the impact of risk factors on psychological distress, especially among immigrants considering their growing numbers in the US.

## 5. Conclusion

Generally, we expect that as people stay longer in a neighborhood, the likelihood of experiencing psychological distress declines as they integrate into the community, assimilate to the culture and norms, socialize, make new friends and families, and network. However, our study found that individuals who reported low neighborhood cohesion experienced higher psychological distress as the length of stay in the neighborhood increased. Additionally, foreign-born individuals experienced increased psychological distress as the duration of their residence in the neighborhood exceeded 10 years. These findings underscore the need to consider how the duration of residence in the neighborhood and other contextual factors may interact to influence psychological distress in a neighborhood as well as overall mental health outcomes of the US population, enabling us to better understand subgroups of individuals at high risk. Overall, decreased neighborhood cohesion was consistently associated with increased psychological distress across all analyses of years lived in the neighborhood. This highlights the importance of adopting strategies to improve neighborhood social cohesion, as it substantially influences residents’ ability to participate in activities, fostering a sense of belonging and social cohesion, which are essential. Our findings also support the development of more effective strategies to create opportunities for interaction and relationship-building within neighborhood communities. Finally, further studies are needed to understand why psychological distress increases in some subgroups of individuals as they live longer in a given neighborhood.

## Figures and Tables

**Table 1:** Sample Descriptive and Bivariate Analysis Stratified by Duration of Residence in Current Neighborhood.

	Total N (%)	≤10 Years in Current Neighborhood n (%)	>10 Years in Current Neighborhood n (%)	Rao-Scott Chi-square p-value
	164,362 (100)	96,175 (58.5)	68,187 (41.5)	
**Variable Description**				
**Psychological Distress (K-6 scale)**				<0.0001
None/Low	129,416 (79.5)	73,735 (77.7)	55,681 (81.9)	
Moderate-Severe	34,946 (20.5)	22,440 (22.3)	12,506 (18.1)	
**Neighborhood Cohesion**				<0.0001
Low Cohesion	52,619 (31.8)	35,432 (36.4)	17,187 (25.4)	
Medium Cohesion	54,107 (33.9)	31,784 (34.0)	22,323 (33.8)	
High Cohesion	57,636 (34.3)	28,959 (29.6)	28,677 (40.8)	
**Nativity/Birthplace**				<0.0001
US-Born	137,897 (82.3)	77,817 (79.1)	60,080 (86.6)	
Foreign-Born	26,465 (17.7)	18,358 (20.9)	8107 (13.4)	
**Covariates Sociodemographics**				
**Sex**				0.4873
Female	88,859 (50.8)	51,930 (50.7)	31,258 (51.0)	
Male	75,503 (49.2)	44,245 (49.3)	36,929 (49.0)	
**Marital Status**				<0.0001
Single/Never Married	37,059 (22.0)	26,689 (24.7)	10,370 (18.3)	
Married/Living with Partner	83,985 (61.3)	47,479 (60.5)	36,506 (62.5)	
Divorced/Separated	15,873 (5.8)	5276 (3.4)	10,597 (9.0)	
Widowed	27,445 (10.9)	16,731 (11.4)	10,714 (10.2)	
**Race/Ethnicity**				<0.0001
non-Hispanic Asian	19,525 (11.1)	12,077 (12.2)	7448 (9.5)	
non-Hispanic Black	8595 (5.6)	5895 (6.5)	2700 (4.3)	
Hispanic/Latino	23,463 (15.4)	16,112 (17.8)	7351 (12.0)	
non-Hispanic White	108,406 (65.7)	59,219 (60.9)	49,187 (72.3)	
Other/Multi-Racial	4373 (2.3)	2872 (2.6)	1501 (1.9)	
**Socioeconomics Education**				<0.0001
Less than High School	17,710 (10.0)	9820 (9.8)	7890 (10.2)	
High School	44,474 (26.9)	23,949 (25.1)	20,525 (29.4)	
Some College	51,137 (31.0)	31,045 (31.6)	20,092 (30.1)	
College/Graduate	51,041 (32.2)	31,361 (33.5)	19,680 (30.3)	
**Employment Status**				<0.0001
Employed	95,456 (62.1)	62,993 (68.3)	32,463 (53.6)	
Unemployed	68,906 (37.9)	33,182 (31.7)	35,724 (46.4)	
**Family Income**				<0.0001
<$25,000	43,249 (18.9)	27,924 (21.4)	15,325 (15.5)	
$25,000-$49,000	40,094 (22.1)	23,336 (22.6)	16,758 (21.2)	
$50,000-$74,000	28,173 (17.9)	15,921 (17.6)	12,252 (18.3)	
≥$75,000	52,846 (41.1)	28,994 (38.3)	23,852 (45.0)	
**Health Behaviors**				
**BMI**				<0.0001
<18.5, Underweight	2870 (1.8)	1733 (1.8)	1137 (1.7)	
18.5–24.99, Healthy weight	54,799 (33.6)	33,933 (35.3)	20,866 (31.2)	
25–29.9 Overweight	56,736 (34.6)	32,226 (33.7)	24,510 (35.7)	
≥30, Obesity	49,957 (30.1)	28,283 (29.2)	21,674 (31.4)	
**Alcohol Drinking Status**				<0.0001
Lifetime abstainer (<12 drinks in life)	31,607 (19.6)	17,406 (18.7)	14,201 (20.7)	
Former (no drinks past year)	25,310 (13.7)	12,462 (11.7)	12,484 (16.5)	
Current (1+ drinks past year)	107,445 (66.8)	66,307 (69.6)	41,138 (62.8)	
**Smoking Status**				<0.0001
Never	98,278 (62.4)	58,952 (63.6)	39,326 (60.8)	
Former	39,295 (22.3)	19,375 (19.1)	19,920 (26.8)	
Current	26,789 (15.3)	17,848 (17.3)	8941 (12.4)	

Note: Data from National Health Interview Survey (NHIS), from 2013 to 2018. Frequency counts are unweighted, and percentages are weighted. Rao-Scott Chi-square test was conducted to examine the differences in moderate-severe PsyD among the levels of exposures and covariates; *p* < 0.05 level of significance implies statistically significant.

**Table 2:** Multivariable Logistic Regression Odds of Moderate-Severe Psychological Distress Stratified by Duration of Residence in Current Neighborhood.

Characteristics	Model I: ≤10 Years in Neighborhood AOR (95 % CI)	Model II: >10 Years in Neighborhood AOR (95 % CI)
**Neighborhood Cohesion**		
Low	**2.01** (1.88 – 2.15)	**2.06** (1.90 – 2.24)
Medium	**1.44** (1.35 – 1.54)	**1.42** (1.31 – 1.55)
High [Ref]	–	–
**Nativity/Birthplace**		
US-Born (Ref)	–	–
Foreign Born	**0.63** (0.45 – 0.82)	1.31 (0.45 – 3.79)
**Covariates**		
**Sociodemographics**		
**Sex**		
Female (Ref)	–	–
Male	**0.73** (0.69 – 0.76)	**0.69** (0.65 – 0.73)
**Marital Status**		
Single/Never Married (Ref)	–	–
Married/Living with Partner	**0.82 (**0.77 – 0.87)	**0.66** (0.61 – 0.73)
Divorced/Separated	1.04 (0.97 – 1.11)	**0.82** (0.75 – 0.90)
Widowed	**0.72**(0.65 – 0.80)	**0.55** (0.49 – 0.61)
**Race/Ethnicity**		
non-Hispanic Asian	1.05 (0.83 – 1.32)	1.07 (0.78 – 1.47)
non-Hispanic Black	0.98 (0.85 – 1.14)	**0.69** (0.57 – 0.83)
Hispanic/Latino	1.13 (0.95 – 1.33)	0.98 (0.81 – 1.18)
non-Hispanic White (Ref)	–	–
Other/Multi-Racial	1.29 (0.97 – 1.71)	0.92 (0.67 – 1.27)
**Socioeconomics**		
**Education**		
Less than High School	**1.27** (1.17 – 1.39)	**1.58** (1.41 – 1.76)
High School	**1.11** (1.04 – 1.19)	**1.22** (1.13 – 1.32)
Some College	**1.16 (**1.10 – 1.23)	**1.26** (1.16 – 1.36)
College/Graduate (Ref)	–	–
**Employment Status**		
Employed (Ref)	–	–
Unemployed	**1.54** (1.47 – 1.61)	**1.34** (1.27 – 1.43)
**Family Income**		
<$25,000	**1.81** (1.69 – 1.94)	**1.95** (1.78 – 2.13)
$25,000-$49,000	**1.39** (1.30 – 1.49)	**1.39 (**1.27 – 1.51)
$50,000-$74,000	**1.21** (1.12 – 1.30)	**1.19** (1.09 – 1.31)
≥$75,000 (Ref)	–	–
**Health Behaviors**		
**BMI**		
<18.5, Underweight	**1.38** (1.19 – 1.61)	**1.30** (1.05 – 1.62)
18.5–24.99, Healthy weight	–	–
(Ref)		
25–29.9 Overweight	1.05 (0.99 – 1.10)	**1.07** (1.00 – 1.15)
≥30, Obesity	**1.35** (1.27 – 1.43)	**1.32** (1.23 – 1.42)
**Alcohol Drinking Status**		
Lifetime abstainer (<12 drinks in life)	–	–
Former (no drinks past year)	**1.42** (1.31 – 1.54)	**1.41** (1.29 – 1.53)
Current (1+ drinks past year)	**1.38** (1.29 – 1.48)	**1.24** (1.15 – 1.34)
**Smoking Status**		
Never (Ref)	–	–
Former	**1.20** (1.14 – 1.27)	**1.11** (1.04 – 1.18)
Current	**1.84** (1.74 – 1.94)	**1.80** (1.66 – 1.96)

Note: Data from National Health Interview Survey (NHIS), from 2013 to 2018; Multivariable logistic regression odds of moderate-severe psychological distress; AOR= adjusted odds ratio; CI= confidence interval; Ref= reference; Bold= statistically significant; *p* < 0.05.

**Table 3:** Interaction between the Years of Residence in Neighborhood and Neighborhood Cohesion and Nativity/Birthplace Status Associated with Moderate-Severe Psychological Distress.

Predictors/Independent Variables	AOR (95 % CI)
**Neighborhood Cohesion**	
Low	**2.12** (1.98 – 2.27)
Medium	**1.47** (1.37 – 1.58)
High [Ref]	**–**
**Nativity/Birthplace**	
US-Born [Ref]	**–**
Foreign-born	**1.12** (1.02 – 1.24)
**Years of Residence in Neighborhood**	
≤10 years	**1.37** (1.29 – 1.49)
>10 years [Ref]	**–**
**Interaction** (neighborhood social cohesion * years of residence)	
Low cohesion * ≤10 years	**0.86** (0.79 – 0.93)
Low cohesion * >10 years [Ref]	**–**
Medium cohesion * ≤10 years	0.93 (0.85 – 1.01)
Medium cohesion * >10 years [Ref]	**–**
**Interaction** (nativity/birthplace * years of residence)	
Foreign-born * ≤10 years	**0.73** (0.66 – 0.81)
Foreign-born* >10 years [Ref]	**–**

Note: Data from National Health Interview Survey (NHIS), from 2013 to 2018; Interaction multivariable logistic regression odds of moderate-severe psychological distress, adjusted for Sociodemographics (sex, marital status, race/ethnicity), Socioeconomic (education, employment, family income), and Health Behaviors (BMI, alcohol status, smoking status); AOR= adjusted odds ratio; CI= confidence interval; Ref= reference; Bold= statistically significant; *p* < 0.05.
